# QuickStats

**Published:** 2013-10-04

**Authors:** Charlotte A. Schoenborn, Patricia F. Adams

**Figure:**
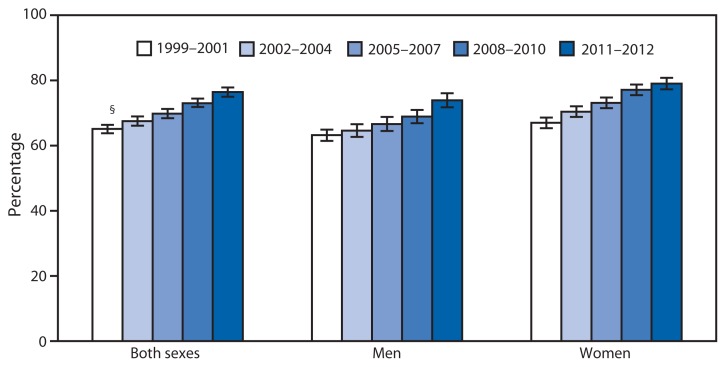
Percentage of Adults Aged 18–24 Years Who Had Never Smoked Cigarettes,* by Sex — National Health Interview Survey, United States, 1999–2001 Through 2011–2012^†^ * Never smoked cigarettes or smoked fewer than 100 cigarettes in lifetime. ^†^ Estimates are annualized averages for each period and are based on household interviews of a sample of the civilian, noninstitutionalized U.S. adult population. Denominator excludes persons with unknown cigarette smoking status. ^§^ 95% confidence interval.

The percentage of young adults aged 18–24 years who had never smoked cigarettes increased by more than 10 percentage points from 1999–2001 (65%) to 2011–2012 (76%). The increase was noted for men and for women. For each period, women were more likely than men to have never smoked cigarettes.

**Sources:** Schoenborn CA, Adams PF, Barnes PM, Vickerie JL, Schiller JS. Health Behaviors of Adults: United States, 1999–2001. Vital Health Stat 2004;10(219).

Adams PF, Schoenborn CA. Health behaviors of adults: United States, 2002–2004. Vital Health Stat 2006;10(230).

Schoenborn CA, Adams PF. Health behaviors of adults: United States, 2005–2007. Vital Health Stat 2010;10(245).

Schoenborn CA, Adams PF, Peregoy JA. Health behavior of adults: United States, 2008–2010. Vital Health Stat 2013;10(257).

National Health Interview Survey. Data and documentation for 2011 and 2012. Available at http://www.cdc.gov/nchs/nhis/quest_data_related_1997_forward.htm.

